# Friction Weldability of a High Nb Containing TiAl Alloy

**DOI:** 10.3390/ma12213556

**Published:** 2019-10-30

**Authors:** Xiangjun Xu, Junpin Lin, Jian Guo, Yongfeng Liang

**Affiliations:** 1 School of Materials Science and Engineering, Yangtze Normal University, Chongqing 408100, China; 2 State Key Laboratory for Advanced Metals and Materials, University of Science and Technology Beijing, Beijing 100083, China; linjunpin@ustb.edu.cn (J.L.); Liangyf@skl.ustb.edu.cn (Y.L.); 3 Materials and Chemistry School, Zhongyuan University of Technology, Zhengzhou 450007, China; 5523@zut.edu.cn

**Keywords:** high Nb containing TiAl, microstructure, friction welding, recrystallization, tensile properties

## Abstract

The friction weldability of Ti-45Al-8.5Nb-0.2W-0.2B-0.02Y alloy has been investigated by optimizing process parameters and analyzing the microstructures and tensile properties of the joints. The as-cast alloy with a nearly lamellar (NL) microstructure and the as-forged alloys with a duplex (DP) microstructure have been successfully welded. All the joints have a severe deformation zone (SDZ) and a transition zone (TZ) between the parent metal (PM) and SDZ. SDZ, showing a biconcave lens geometry, has a maximum thickness of hundreds of micrometers at the periphery. TZ is hundreds of micrometers thick. All SDZs have a fine-grained DP microstructure with a grain size of a few micrometers. For the joint of the as-cast alloy, the TZ consists of deformed lamellar colonies as the major constituent and partially recrystallized grains as the minor constituent. For the joint of the as-forged alloy, the TZ is similar to both the PM and SDZ, showing a DP microstructure. The grain size, volume fraction of γ grains, and the remnant lamellar colonies all increase with the distance from the SDZ. All joints presented perfect metallurgical bonding. The strengths of the joints are higher than those of the corresponding PMs. This indicates that the studied alloy has good friction weldability.

## 1. Introduction

Due to their attractive properties such as low density, good elevated temperature strength, high specific stiffness, and excellent creep properties, TiAl-based alloys have been regarded as ideal high-temperature structural materials in aerospace and automotive industries [1,2,3,4]. Nb addition can increase the elevated temperature properties of TiAl alloys, especially for oxidation resistance; thus, high Nb containing TiAl alloys have become a kind of promoting high-performance alloy [5,6,7]. Some high Nb containing TiAl alloys have been applied for automotive engine parts. For example, the Ti-46/47Al-6Nb alloy has been designed for automotive engine turbocharger wheels at 1100 °C and Ti-44Al-6Nb-0.3W-0.1B alloy has been used for race vehicle engine exhaust valves at temperatures above 800 °C [8]. The Ti-45Al-8.5Nb-0.2W-0.2B-0.02Y alloy is also of great potential to be widely applied in these areas because of its good properties. 

Welding is a basic process for connecting the turbo shaft and turbo wheel, as well as connecting the head and stem of the exhaust valve. Although TiAl joints have been obtained by means of numerous approaches, solid state joining processes including brazing, diffusion bonding, and friction welding are more suitable processes because TiAl alloys are more sensitive to thermal stress. Ti-based and Ag-based filler metals are the most commonly used for the brazing of TiAl alloys [9]. This can change the composition and properties of the alloys. In the diffusion welding process, oxygen and nitrogen contamination of the mating surfaces cannot be avoided [10]. Rotary friction welding (RFW) is one of the best processing techniques for joining these axisymmetric TiAl parts [11], because neither the filler material nor any inert gas is needed to ensure the welding joint is uncontaminated with the same composition as the parent metal (PM) [12]. Many works investigating the friction welding of conventional TiAl have been performed in the past years. For example, in 1993 Miyashita et al. welded as-cast Ti-48Al alloy rods with diameters of 6 and 12 mm [13]. In 1996 Shinoda et al. welded Ti-49Al-1Mn alloy rods with a diameter of 15 mm [14]. In 2003 Bohm et al. reported the result of welding Ti-47Al-3.5(Mn+Cr+Nb)-0.8(B+Si) alloy [15]. Recently, Sankar et al. reported the result of welding Ti-48Al-2Cr-2Nb alloy [16]. However, the related work on high Nb containing TiAl alloy has not been reported. In the present paper, the friction welding parameters of Ti-45Al-8.5Nb-0.2W-0.2B-0.02Y alloy was studied by trial welding, and the as-cast and as-forged alloys were successfully welded by an optimized RFW process. The microstructures of the welding joints were characterized. To evaluate the weldability of the studied alloy, the strengths of the joints were also measured. 

## 2. Experimental

### 2.1. Materials

The nominal composition of the material is Ti-45Al-8.5Nb-0.2W-0.2B-0.02Y (in at %). An ingot with dimensions of 760 mm × 380 mm × 900 mm was prepared by a four-torch plasma arc cold hearth melting furnace. A cylindrical billet with dimensions of Φ130 mm × 150 mm was cut from the ingot and then canned in a stainless-steel jacket and forged by a two-step forging process. The forging was carried out at about 1250 °C with a strain rate of 1 × 10^−3^ s^−1^ and a reduction of 80%. Rods with dimension of Φ10 mm × 40~100 mm were cut from the ingot and the forged pancake, respectively, by electrical discharge machining (EDM). The as-cast alloy was annealed at 1000 °C for 5 h. All rods were roughly ground to eliminate the oxidized skin and the cutting marks by emery paper.

### 2.2. Selection and Optimization of Welding Parameters

Rods were welded by a continuous drive friction welding machine model MCH-5SJ (Shanghai Shengchun Machinery Co., Ltd, Shanghai, China). The adjustable parameters are rotation speed (ω), friction pressure (P_f_), friction burn-off distance (δL), upset pressure (P_u_), and upset rate ($\dot{\varepsilon }$). Trial welding is necessary to fix the workable settings for all of the variables. Firstly, eight pairs of rods from the as-cast alloy were used as trial weld specimens, which were welded by the parameters given in Table 1. The reasons for choosing these schemes will be explained in the next paragraph. Based on the appearances of the welding joints and the metallographic structures of the trial welds, the final parameters employed of ω, P_f_, δL, P_u_ and $\dot{\varepsilon }$ were 2400 r/min, 320 MPa, 3 mm, 360 MPa, and 4 mm/s, respectively. The reason will be given in Section 3. Then, six pairs of rods, three pairs of as-cast alloy, and three pairs of the as-forged alloy were welded using the final parameters.

The idea of developing the trial welding schemes is given in detail in this paragraph. The heat flux density generated by friction welding on the abutting surface is:

q_0_/A=2/3 μP_f_ v_max_ (1)

where μ, P_f_ and v_max_ are the friction coefficient, friction pressure, and maximum linear velocity of rotation, respectively [17]. Therefore, in the case of a constant composition and size of the specimens, increases of P_f_ and ω result in higher heat in a short time. For high efficiency, we chose a ω of 2400 rpm. This speed is a relatively large value, as the maximum speeds of many devices were less than 2000 rpm. In order to ensure the dimensional tolerance of welding parts and also improve the welding efficiency, shorter friction time and higher friction pressure were preferable. The friction time was set through δL, and two values of 2 mm and 3 mm were chosen (Schemes 2 and 5 were 2 mm, and the other schemes were 3 mm). The P_f_ selected in this study ranged from 240 MPa to 360 MPa. For the 2 mm δL schemes, P_f_ of 240 MPa (Scheme 2) and 280 MPa (Scheme 5) were selected, and for the 3 mm δL schemes, P_f_ of 240 MPa (Schemes 1 and 3), 280 MPa (Scheme 4), 320 MPa (Schemes 6 and 7) and 360 MPa (Scheme 8) were selected. In order to extrude all the original materials from the friction surfaces so as to avoid contamination of the abutting surfaces and obtain sound welds, larger upset force is favorable. The P_u_ selected varied from 280 MPa to 360 MPa. For the 2 mm δL schemes, P_u_ of 280 MPa (Scheme 2) and 320 MPa (Scheme 5) were selected, and for the 3 mm δL schemes, P_u_ of 280 MPa (Schemes 3 and 4), 300 MPa (Scheme 1) and 360 MPa (Schemes 6–8) were selected. Given the brittleness of the material, a lower upset rate of about 4 mm/s was used in most cases, and a higher upset rate of about 20 mm/s was only applied in one case (Scheme 7). The welding parameters used and the overall reductions of the specimens (ΔL) are shown in Table 1.

### 2.3. Macro- and Microstructure and Tensile Properties Analyses

After welding, the joints were bisected longitudinally by EDM. The sections were mechanically ground and electrolytically polished to prepare the metallographic samples, and then were analyzed by a Zeiss LSM700 confocal laser microscope (CLSM, Braunschweig, Germany) and the Zeiss Supra55 or Regulus 8100 scanning electron microscope (SEM, Braunschweig, Germany and Tokyo, Japan, respectively) in backscattered electron (BSE) mode. The electropolishing was conducted using a solution of 5% perchloric acid, 35% butanol, and 60% methanol at −20 °C under 30 V. For CLSM analysis, the samples were etched by a Kroll’s reagent. The joint of as-cast alloy was additionally examined by a Tecnai G2 F30 field emission transmission electron microscope (TEM, Hillsboro, OR, USA). TEM thin foils were machined from the joint and prepared by standard twin-jet electrochemical polishing. The electrolyte was the same as that for metallographic samples. 

Room temperature tensile tests were conducted on cylindrical specimens, which were prepared by mechanical processing of welded rods. The gauge section of each specimen was Φ 3.5 mm × 25 mm with the joint in the middle of it. The tests were conducted using a 50 kN model DDL50 universal testing machine (Zhuhai Sust Electrical Equipment Co., Ltd., Zhuhai, China) at a strain rate of 5 × 10^−4^ s^−1^. The data collected were force and displacement. Two specimens were tested for each kind of joint, and the average strength was calculated. The fracture surfaces of the specimens were observed via secondary electron imaging in a SEM.

## 3. Results and Discussion

### 3.1. Microstructures of the As-Cast and As-Forged Alloys 

**Figure 1:**
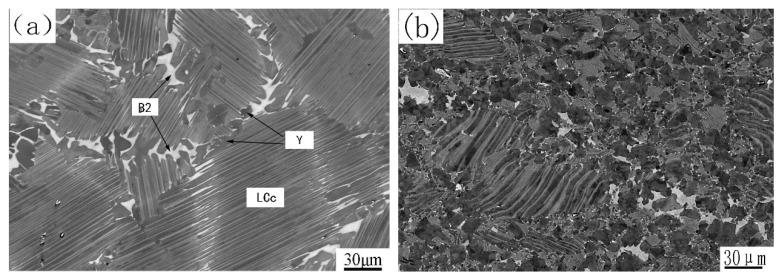
Microstructures of the as-cast alloy (**a**) and the as-forged alloy (**b**).

Figure 1a shows the as-cast microstructure. It is a nearly lamellar (NL) microstructure, in which lamellar colonies (marked as LCc) are the major constituent. The minor constituents such as B2 and γ grains are mainly located at colony boundaries or triple junctions. The volume fractions of the three constituents are about 83%, 7% and 10%, respectively. The average lamellar colony size of LCc is about 150 μm, and both the grain sizes of B2 and γ phases are less than 20 μm. Theoretically, B2 phase should not exist in the as-cast alloy for the microstructure formed according to the following solidification and transformation path: L→ β → β + α → α → α_2_ + γ. However, during L → β solidification, the primary β phase formed dendrites. Element partition determined that interdendritic zones contain higher Al content and the spines of the dendrites are enriched by β-formation elements, such as Nb and W. Because in the Ti-Al-Nb phase diagram, α phase is at the Al-rich end of the tie-line of α and β phases, and Nb and W tend to concentrate in the β phase and stabilize the β phase [18], during β → β + α → α transformation, Al diffused to α phase while Nb and W diffused in the opposite direction. This led to an enrichment of Nb and W and the depletion of Al at the interfaces between β and α grains. These Nb-rich zones transformed via a β → B2 + γ path. So, B2 is a metastable phase resulting from microsegregation, which was induced by element distribution during solidification and transformation. 

Figure 1b shows the as-forged microstructure, which mainly exhibits duplex (DP) microstructure consisting of recrystallized γ grains, recrystallized lamellar colonies and some retained B2 phase grains. The mean grain size is about 15 μm, and the volume fraction of B2 phase is about 4%. Some remnant lamellar colonies, with volume fraction of about 20%, can also be observed. However, these lamellar colonies have a significantly smaller size and often exhibit bent lamellae. These colonies resulted from insufficient recrystallization, so we call them remnant lamellar colonies. At 1250 °C the point of alloy composition is located within the γ + α phase region [19]. Canned forging at this temperature resulted in lamellar colonies buckling as well as plastic deforming and shearing. The localization of strain led to a significant refinement of the grain size due to dynamic and static recrystallization, thus most of LCc were replaced by the finer γ + α grains. DP microstructure formed during cooling by prior α grains transforming into finer lamellar colonies. The degree of recrystallization of LCc was determined by their deformation. LCc with soft orientation, especially those located at the near surface and the rim zones of pancake, were partly broken up, forming remnant lamellar colonies surrounded by the DP microstructure. Forging also intermixed regions of different compositions and reduced the diffusion distances of elements. The stress and temperature also provided sufficient energy to improve the diffusion rate of W and Nb atoms. Thus, these elements could overcome the activation barrier to decrease the extent of segregation and to decompose some metastable B2 phase. So, the as-forged microstructure has a finer grain size and less metastable B2 phase compared with the as-cast microstructure.

### 3.2. Joints of the Trial Welding Specimens

#### 3.2.1. The Appearances 

**Figure 2:**
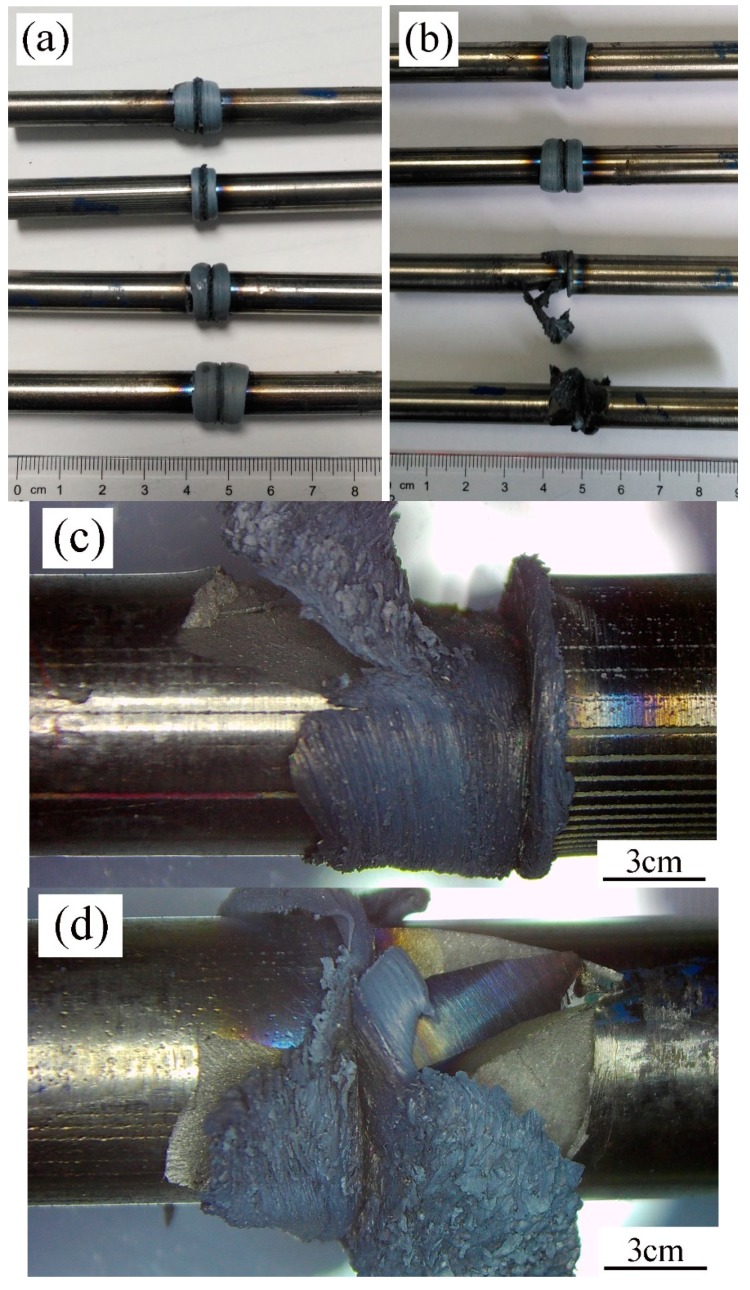
Appearances of the joints of the trial welding specimens: (**a**) Schemes 1–4; (**b**) Schemes 5–8; (**c**) cracking occurring at upset stage in Scheme 7; (**d**) cracking occurring at friction stage in Scheme 8.

Figure 2 shows the appearances of specimens obtained by trial welding processes. Figure 2a is the results of Schemes 1 through 4 from top to bottom, while Figure 2b is the results of Schemes 5 through 8 from top to bottom. It can be seen that the joints with perfect appearance could be obtained when δL was 2 mm or 3 mm, ω was 2400 rpm, P_f_ was 240–320 MPa and P_u_ was 280–360 MPa, and upset rate was 4 mm/s. The welding flash formed on the two sides of the contact surface of each joint, which is discerned by the narrow gap between the flash. No cracks or even microcracks were found on the surface. The size reduction of the specimen (ΔL), which was mainly induced by the formation of flash, increases with the increase of δL and P_f_. For example, Schemes 2 and 3 have the same P_f_, 240 MPa, and the same P_u_, 280 MPa, but different δL, which are 2 mm and 3 mm, respectively. This resulted in the two different ΔL of 2.35 mm and 4.21 mm, respectively. ΔL includes δL and dL (upset distance), so in these two cases the dL are 0.35 mm and 1.21 mm, respectively. This indicates that the longer friction burn-off distance makes the specimen have a larger area of softening metal. As another two examples, Schemes 2 and 5 have the same δL, 2 mm, but the P_f_ and P_u_ in Scheme 5 are larger than those in Scheme 2, leading to the two ΔL of 2.35 mm and 3.64 mm, respectively. The dL in these two cases are 0.35 mm and 0.64 mm, respectively. Similarly, Schemes 3, 4, and 6 have the same δL, 3 mm, but increased P_f_ and P_u_. This led to three different ΔL, 4.21 mm, 4.50 mm, and 4.46 mm, and three different dL, 1.21 mm, 1.50 mm, and 1.46 mm. The reasons for these results are given as follows.

During the friction stage the heat generated increased, thus the temperature of the friction surface rose, and the length of heated zone increased. When the heat generated by the fraction was equal to that transferred by the flash and the chucks, a thermal equilibrium was established, and a dynamic equilibrium between the worn out high temperature zone and the newly formed high temperature zone was established near the contact surface, leading to a constant length of softened metal. To establish this dynamic equilibrium, a sufficient friction burn-off distance and friction pressure are required. The dL produced at each P_u_, i.e., the amount of metal that squeezed out as a flash, almost depends on the length of the heated and softened metal. Under the condition that this length is constant, and that P_u_ is not much different, dL is almost invariant. This can also be seen by the metallographic analysis of weld metal in the next section. So, under the conditions of P_f_ > 280MPa and δL being 3 mm, a stable heated length can be formed. An increase of δL will not make much sense, except to consume PM, increase flash, and reduce welding efficiency. 

Scheme 7 is a case in which the joint is crushed during the upset stage, shown by cracking of the abutting end of a rod and flying away of the debris (Figure 2c). The difference between Scheme 7 and Scheme 6 is only the upset rate, which are 4 mm/s and 20 mm/s, respectively. This is attributed to the train rate sensitivity of this alloy. The upset stage is regarded as a forging process of the joint in which lower temperature parts of two rods take a part of the flat die. However, a lower strain rate is necessary to forge high-Nb containing TiAl alloys [20], so the upset rate in Scheme 7 was so high that the compressive strain rate exceeded the maximum allowable strain rate for forging the softening metals of joint, resulting in cracking. Scheme 8 adopted the largest P_f_ value in the study. In the early stage of friction, lesser heat was produced, and the abutting ends of the rods were in a brittle state. Because the resultant stress of P_f_ and tangential stress generated by friction exceeded the strength of the abutting ends, cracking occurred, and the welding process was stopped by the emergency stop (Figure 2d). From Schemes 7 and 8, it is concluded that thermal equilibrium should not be established under large frictional stress and a slower upset rate is necessary to avoid cracking.

#### 3.2.2. Macrostructures of the Welding Joints

**Figure 3:**
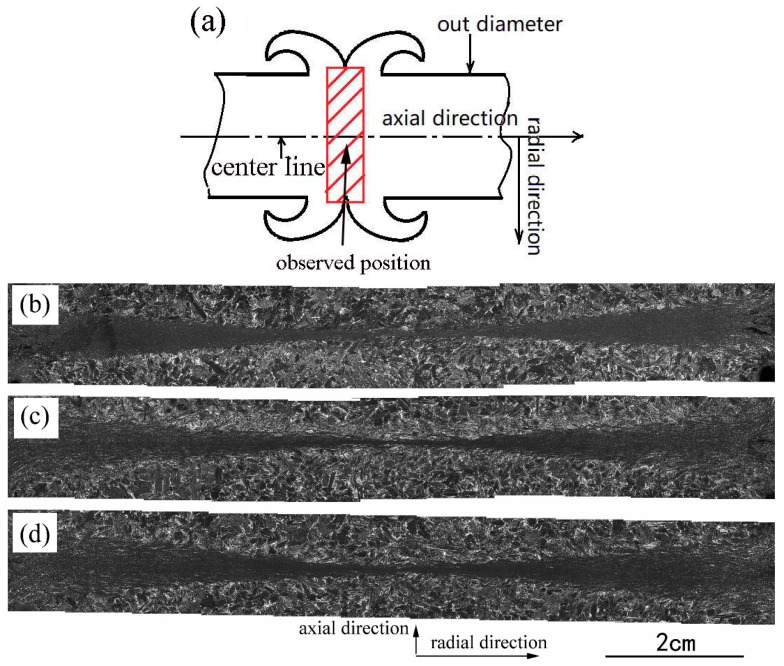
Macrostructures of the joints: (**a**) schematic diagram of observed position; (**b**) joint of Scheme 2; (**c**) joint of Scheme 5 and (**d**) joint of Scheme 6.

Figure 3 shows the macrostructures of the longitudinal sections of the welded joints obtained by Schemes 2, 5, and 6. It can be clearly seen that the insides of all welds are completely free of any voids and cracks, and that a complete metallurgical bond can be obtained by these schemes. For joints obtained by Schemes 5 and 6, they each have two characteristic zones, a severe deformation zone (SDZ) and PM, without any obvious structure gradient between them. However, for the joint obtained by Scheme 2 the distinction between SDZ and PM is not so obvious near the axis of the specimen. The boundary between SDZ and PM reflects different reactions to the etchant of different microstructures, which indirectly shows the temperature distribution in the joint. The SDZ is the higher temperature and softening zone, showing a form of symmetrical double concave lens, i.e., the joint has the shortest and longest heated length at the central axis and the outer diameter of the welded specimen, respectively. This is because the heat generated by friction is proportional to the linear speed of the contact surface [17]. For the rods the periphery has the highest velocity and thus generated the most heat. By contrast, the axis of the rod has a linear velocity of zero, and theoretically no heat was generated. The heat in the center line was mainly transferred by the surrounding metal through heat conduction. For each joint the thinnest and the thickest heated zone are less than 100 μm and 1000 μm, respectively. This indicates that the cooling effect limited the temperature at the contact surface and its vicinity, and the temperature drastically decreased with the increase of axial distance from the contact surface. So, the temperature distribution along the axial direction across the contact surface shows a “λ” profile [21]. The localized heating is also shown by the length of thermal tinting shown in Figure 1. 

The macrostructures of welds obtained by Schemes 5 and 6 are relatively similar, which reflects that the heated length does not change much under the condition of thermal equilibrium. This corresponds to the similar size of the flashes of the two specimens shown in Figure 1. On the contrary, a thermal equilibrium had not been established in Scheme 2, which makes the softening area too short at the centre of the sample, resulting in less deformation.

### 3.3. Optimized Process and Welding Results

According to the appearances and macrostructures of the joints obtained by trial welding, it is concluded that through a reasonable combination of friction pressure and friction burn-off distance, the weld can be heated at a moderate rate and a relatively stable heating zone can be obtained. Then, a dense and non-cracking joint can be obtained by upsetting it under a pressure slightly higher than that of the fiction at a lower upset rate. After comprehensive consideration of welding efficiency, dimensional tolerance and the reliability of the welding joint, a process of rotation speed of 2400 r/min, δL of 3 mm, P_f_ of 320 MPa, P_u_ of 360 MPa, as well as an upset rate of 4 mm/s, was applied to weld the as-cast and as-forged alloys for further characterization.

**Figure 4:**
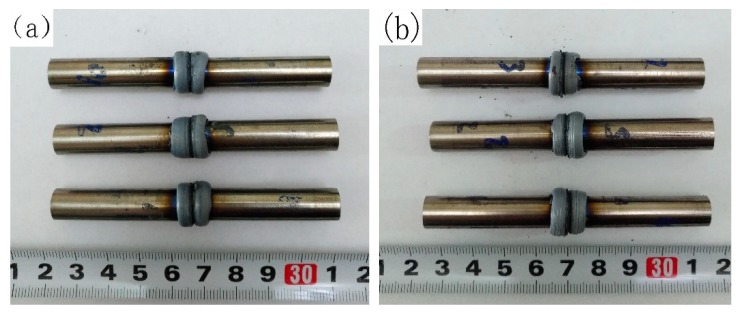
Welded joints of the as-cast alloy (**a**) and the as-forged alloy (**b**).

Figure 4a,b shows welded specimens of the as-cast alloy and the as-forged alloy, respectively. The good appearances of the welding joints indicate: (1) Ti-45Al-8.5Nb-0.2W-0.2B-0.02Y alloy with both as-cast and as-forged microstructures is not susceptible to the thermal stress formed during friction welding; (2) using the optimized process, the reproducibility of welding results is very good. 

### 3.4. Structure of the Specimens Welded by the Optimized Process

#### 3.4.1. Macrostructures

**Figure 5:**
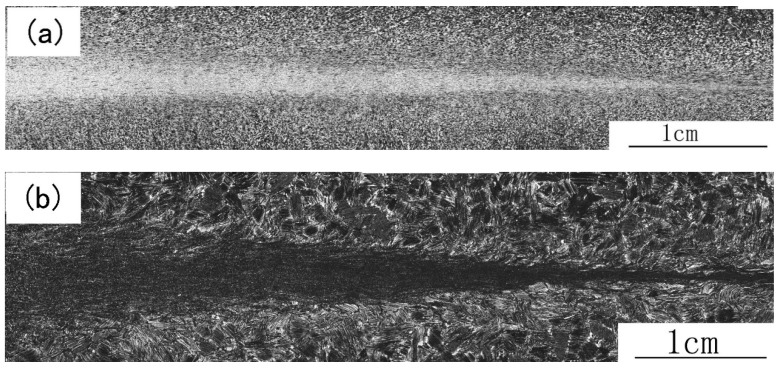
Macrostructures of the welded joints: (**a**) the as-forged alloy; (**b**) the as-cast alloy.

Figure 5a shows the macrostructure of half of the welding joint of the as-forged alloy (marked as FJ). For comparison, the macrostructure of as-cast alloy (marked as CJ) is shown another time in Figure 5b. The sampling method is the same as that shown in Figure 3. Because of symmetry, only half of each joint is shown here.

Both the macrostructures show the metallurgical integrity of the joints. These results are similar to those reported in References 13, 14 and 16. From the appearances and the macrostructures, it can be preliminarily inferred that the present alloy has good weldability. In contrast with these results, the quality of friction welding joints of Ti-47Al-3.5(Mn+Cr+Nb)-0.8(B+Si) alloy is not so good. It is possible to detect some lack of fusion and an extremely high number of pores in the welding joints. Smoothing the contact surface by grinding and polishing can eliminate the lack of fusion, but occasional microspores with a diameter of 1–2 μm were found in the periphery region of the joint zone [15].

Figure 5 also shows that the thicknesses of the central and peripheral zones of SDZ of CJ are about 40 μm and 800 μm, respectively, and the corresponding values of FJ are about 50 μm and 500 μm, respectively. This reflects different dynamic recrystallization processes in different microstructures. During the friction and upset stages, the softening metal at the contact surface was forced radially outward to form the flash, the newly formed contact surface bonded, and its immediate vicinity with various thicknesses underwent recrystallization. For Ti-45Al-8.5Nb-0.2W-0.2B-0.02Y alloy with as-cast microstructure distinct recrystallization occurs at temperature above 1100 °C, so the narrow partial recrystallization zone, which is about 900–1100 °C, cannot be discerned in the macrograph [20]. For the as-forged microstructure though the recrystallization may occur at lower temperature, partial recrystallization zone is also too narrow to be discerned at lower magnification owing to the microstructural similarity between this zone and the PM. The metallurgical integrity of both CJ and FJ indicates that the welding parameters are reasonable to properly establish thermal and mechanical conditions at the weld zones. 

#### 3.4.2. The Microstructure of the CJ

**Figure 6:**
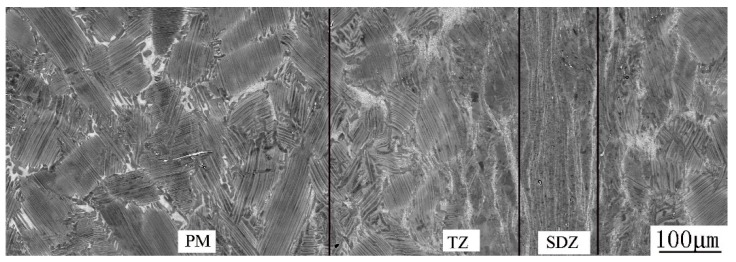
Microstructure evolution from PM to SDZ along axial direction of the CJ.

Figure 6 shows the microstructure evolution from PM to SDZ along the axial direction of the CJ. SDZ, which is obviously distinguished by the typical flow lines of forging, is composed of alternate white, gray, and dark bands parallel to the contact surface. It is worthy to note that no bond interface exists in the SDZ, so the sound bond is also verified by observation at high magnification. This is in agreement with the results of others [13,14,16]. In diffusion bonded TiAl alloys, the bond interface was clearly visible due to the discontinuity of the grains in both sides of each join, and the α_2_ phase formed in the bond interface because of oxygen and nitrogen contamination of the contact surface [10]. During friction welding when the hot interface material contacts the atmosphere at the initial friction step, oxygen and nitrogen contamination cannot be completely avoided [22]. However, the friction and upset loads (on the order of 200 MPa) are more than ten times higher than the loads applied to the diffusion bonded joints (on the order of 10 MPa). Therefore, the welds are free from contaminants because all of the initial contact interface materials are expelled into the flash [22]. The PM is an NL microstructure as shown earlier. There exists a transition zone (marked as TZ) with a thickness of about 300–400 μm between the SDZ and PM, which is characterized by lamellar colonies with slightly curved lamellae, blurred lamellar interfaces and colony boundaries. After observing them by SEM and TEM at higher magnifications, we expound the characteristics and formation mechanisms of SDZ and TZ as follows.

**Figure 7:**
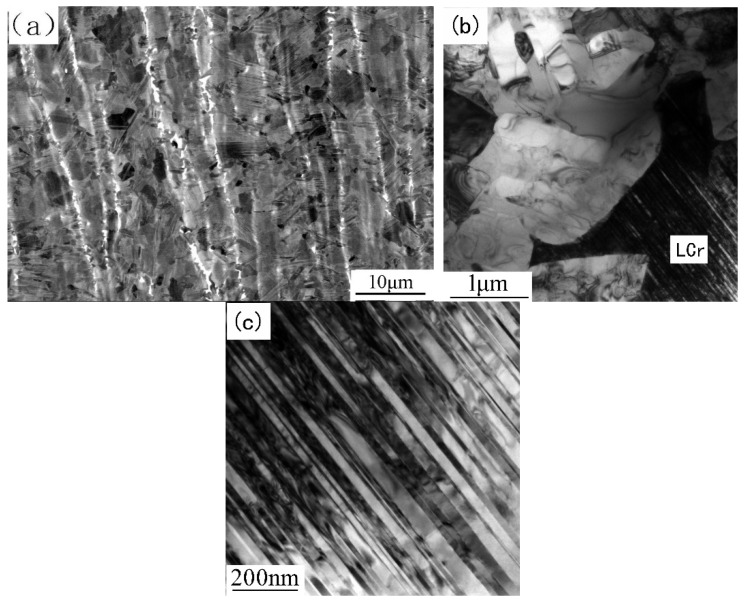
Microstructure of SDZ in CJ: (**a**) SEM-BSE image; (**b**) TEM image; (**c**) TEM image showing the thinner lamellar spacing of the LCr.

A SEM-BSE image of SDZ at higher magnification is shown in Figure 7a. The dark and gray bands are fine grained DP microstructure with different Al content and the white bands are B2 grains enrichment zones. The average grain size of the SDZ is several micrometers, which is much smaller than that of the as-forged microstructure. The grain size distribution is very heterogeneous, and the fine recrystallized lamellar colonies (marked as LCr) have a larger grain size than γ and B2 grains. The volume fraction of B2 phase is about 3%, which is lower than that in PM. A TEM image clearly revealing the substructure of SDZ is shown in Figure 7b. There exists high density dislocation and many subgrains in the γ grain, showing the occurrence of complex recovery and recrystallization. The lamellar spacing of LCr is much thinner than those of the lamellar colonies in both as-cast and as-forged alloys (Figure 7c).

The fine-grained DP microstructure in the SDZ is an indication that complete dynamic recrystallization takes place at the α + γ phase region. This is similar to the formation of the as-forged microstructure. However, the finer grains are attributed to the severe deformation of the metal close to the contact surface. The flow line pattern apparently shows that compressive strain occurs in the axial direction and tensile strain occurs in the radial direction. It is assumed that B2 phase has a relatively low yield strength at higher temperatures, so B2 phase deforms more easily than α and γ phases during the upset stage. As a result, the B2-rich zones were pressed into nearly continuous flakes for many of them originally belonged to the same β dendrite. However, the volume fraction of B2 phase also decreased, with the mechanism just like the as-forged microstructure. The regions with different Al contents were also compressed in the axial direction and extended in the radial direction to transform into different DP bands. This banded structure is different from the as-forged microstructure. The as-forged structure was formed by two steps, and the composition of different parts was fully mixed. Because of the short welding time, the fully recrystallized grains had no chance to further grow up before cooling. Consequently, fine-grained DP microstructure with a sandwich morphology was formed in SDZ. The relatively fast cooling rate also resulted in the recrystallized α forming LCr with a thinner lamellar spacing. However, during friction welding, dynamic recrystallization was a continuous process. The nucleation of grains and subsequent migration of grain boundaries led to new dislocation-free grains, but further deformation and nucleation may occur within these grains. So, the grain size and dislocation distribution are very heterogeneous.

**Figure 8:**
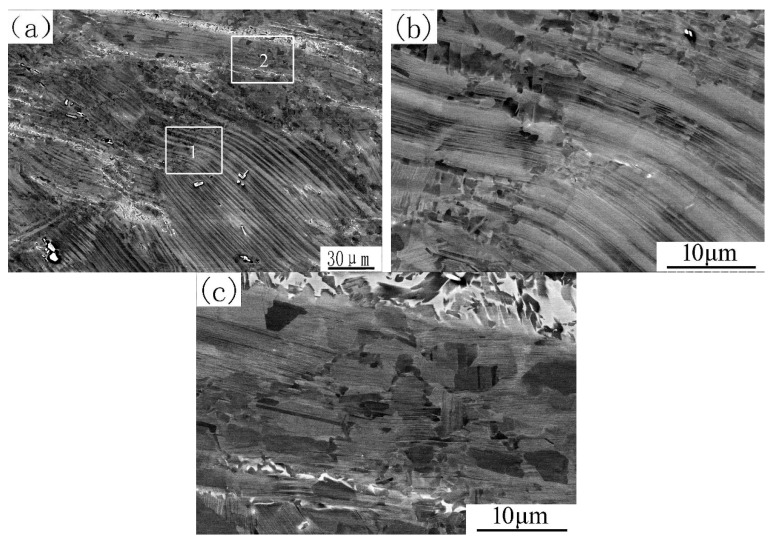
Microstructure of TZ close to SDZ: (**a**) low magnification image; (**b**) and (**c**) magnified images of areas 1 and 2 in (**a**), respectively.

The microstructure of TZ varies from place to place. Near the SDZ, it is evident that large deformation and partial recrystallization are dominant characteristics. The lamellae are curved upon limited compressive strain. Some areas within the initial LCc or at the colony boundaries were completely broken down into globular microstructure, but the bulk of initial LCc still exhibits the lamellar morphology at low magnification (Figure 8a). One reason for this is that, although fine recrystallized grains were formed within the LCc, they aligned along the direction of the lamellae, forming a chain morphology. This phenomenon is shown in Figure 8b, which is a magnified image of the square area 1 in Figure 8a. Another reason is that many new LCr had the orientation similar to that of the parent LCc. This phenomenon is shown in Figure 8c, which is a close-up image of the square area 2 in Figure 8a. From this, one can draw the conclusion that formation sequence of the LCr is LCc → α → recrystallized α → LCr and some recrystallized α grains nearly keep the orientation of α coming from LCc. It is worthy to note that the retained lamellar colonies have an extremely thin lamellar spacing, contrasting sharply with the LCc shown in Figure 1. This can also verify the occurrence of LCc → α transformation, after which, new fine α grains recrystallized in the large deformation zone. Both the new recrystallized α grains and the non-recrystallized α colonies were subjected to a rapid cooling, making all the lamellar colonies have very thin lamellar spacing.

**Figure 9:**
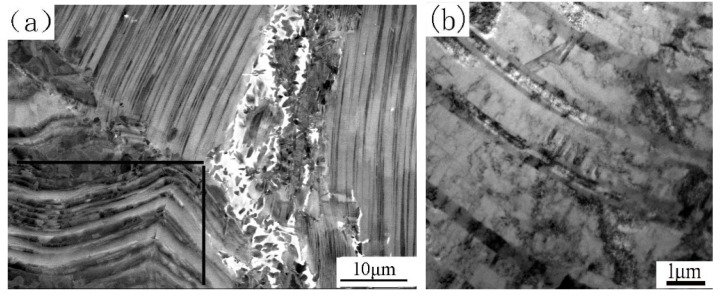
Microstructure of TZ near PM: (**a**) SEM-BSE image and (**b**) TEM image.

In TZ, close to PM, some γ lamellae thickened, and the interfaces of the lamellae are blurred. Fine recrystallized grains were formed in the thick lamellae, as shown in the square area in Figure 9a. The γ and B2 phases at triple junctions of LCc transformed into finer γ, α_2_ and B2 grains. This conforms to the B2 degradation mechanism previously found by our group: Holding the as-cast alloy at medium temperature, a part of the B2 phase directly transforms to γ phase, holding at high temperature, most of the B2 phase directly transforms to α_2_ phase [23]. These triple junction zones have been subjected to a wide range of temperatures from the room temperature to temperature of α + γ phase region, so both degradation mechanisms take effect. The relatively low strain imparted during welding made the crystallization process superimpose on the degradation process of B2 phase, encouraging the formation of finer γ and α_2_ grains in this short welding time. For some LCc near PM, though no distinct recrystallization was observed under SEM, and microstructural change can be clearly identified under TEM. Figure 9b shows the substructure of a slightly deformed lamellar colony. Some curved lamellae with blurred lamellar interfaces can be observed. So, a strict Blackburn orientation relationship between γ and α_2_ lamellae and the three relationships between γ lamellae in a perfect lamellar structure have been changed by the high temperature and deformation. High-density dislocation and twins formed in γ lamellae. Many lamellar interfaces are disappearing since the interconversion between γ and α_2_ phase occurred in the lamellar structure. This indicates that the lamellae have undergone a complex process including deformation and growth. So, for LCc near PM, the newly recrystallized grains, the intersecting twin, and the lamella growing into adjacent lamella led to the blurred lamellar interfaces. 

#### 3.4.3. The Microstructure of FJ

**Figure 10:**
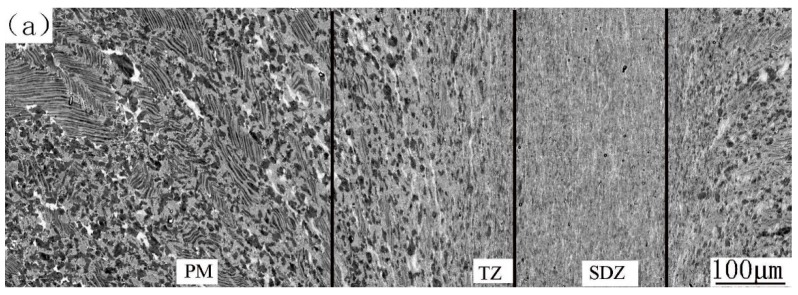

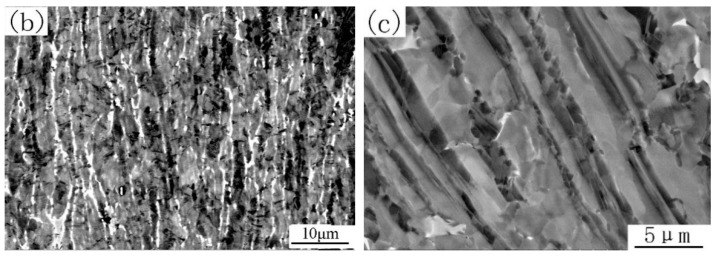
Microstructure of FJ: (**a**) microstructure evolution from PM to SDZ; (**b**) SDZ; (**c**) recrystallization in remnant LCc.

There are also three zones in FJ (Figure 10a). Faint flow lines without black bands show the deformation of the SDZ. Actually, the black bands do not exist in the as-forged microstructure (the PM of FJ) either. This indicates that Al segregation in the ingot can be eliminated by forging at high deformation ratio. So, the SDZ of FJ inherited the homogeneous composition of the as-forged alloy. High magnification images show that the microstructure of the SDZ of FJ is similar to that of the SDZ of CJ in both grain size and the volume fraction of B2 phase (Figure 10b). That is, the microstructure of SDZ of FJ is also a fine DP microstructure containing a few B2 phases. The microstructure from SDZ to PM transits smoothly. There also exists a TZ, although the boundaries between SDZ and TZ and those between PM and TZ are not distinct. In TZ the evolution trend of microstructure is that the grain size, the volume fraction of γ grain and the volume of the remnant lamellae all increase with the increase of the distance from SDZ. This is also because the deformation ratio and the temperature gradually decrease with the distance departing from the contact surface. Only partial recrystallization occurred in the TZ, leading to a decreased grain size compared with the as-forged PM. It is inferred from the microstructure that during upset stage the temperature of this TZ was mainly located within α + γ phase region. Since the temperature decreases from SDZ to PM, γ fraction must increase. This is easy to understand from Ti-Al and Ti-Al-Nb phase diagrams [19]. The decrease of remnant lamellar colonies is also attributed to the recrystallization, which cannot break up all the remnant lamellae completely, especially those in TZ close to PM. A high magnification image of these remnant lamellae in TZ is shown in Figure 10c. Nearly complete recrystallization occurred, and many fine grains formed in each lamella. But the recrystallized grains aligned along the direction of original lamellae, indicating in this way the locus of the original lamellae. It is possible that more deformation can disorganize this arrangement and results in grains of random orientation. This phenomenon shows that a transformation from an NL microstructure to a fine-grained DP microstructure of random orientation is a result of multiple recrystallization and high deformation processes.

### 3.5. Tensile Properties and Weldability Assessment

#### 3.5.1. Tensile Properties of the Joints

**Figure 11:**
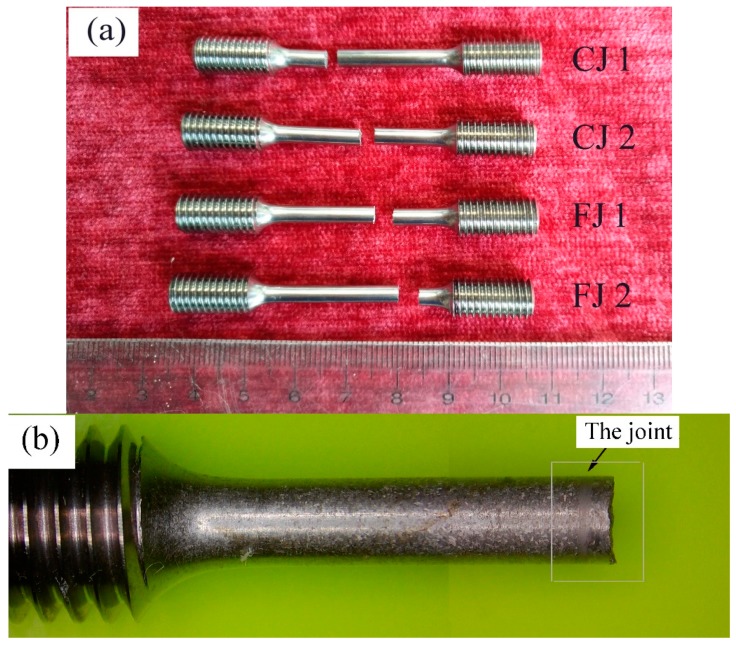

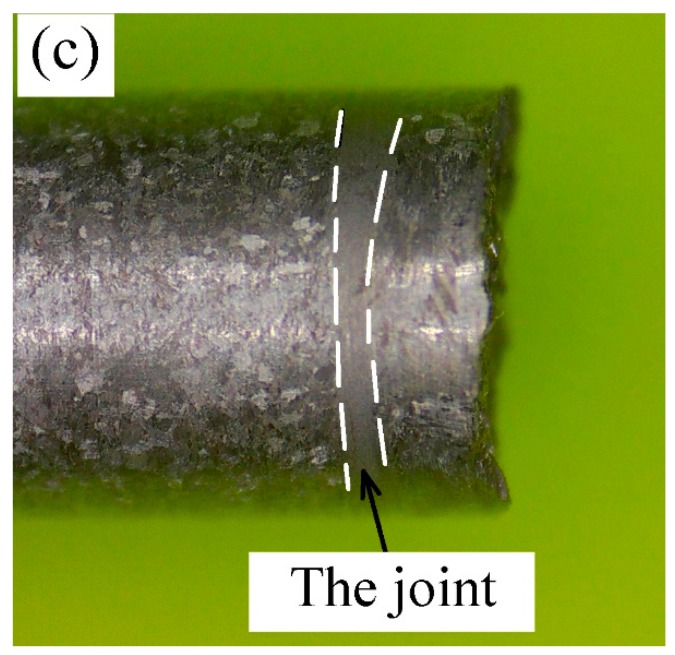
Photographs of the fractured tensile specimens: (**a**) all specimens; (**b**) the left half of CJ2; (**c**) close-up of the joint.

Figure 11a shows the fractured tensile specimens. The fracture surfaces are relatively flat and nearly perpendicular to the direction of the applied tensile stress. No necking and other plastic deformation signs occurred in the gauge sections of all specimens. These characteristics obviously show that the specimens failed in a brittle manner. All the specimens except CJ2 are found to fail in the PMs far away from the welding joints (the centers of the specimens). When the surface of CJ2 is etched by Kroll’s reagent, it is found that the welding joint is at the left half of the fractured specimen. Figure 11b, c shows the magnified image of the left half of the CJ2 and a close-up of its weld. Although the contrast is weak under macroscopic observation, it can still be seen that the grain of the PM is coarse while that of the weld is relatively fine. Thus, all the joints have greater strength than the PMs.

**Figure 12:**
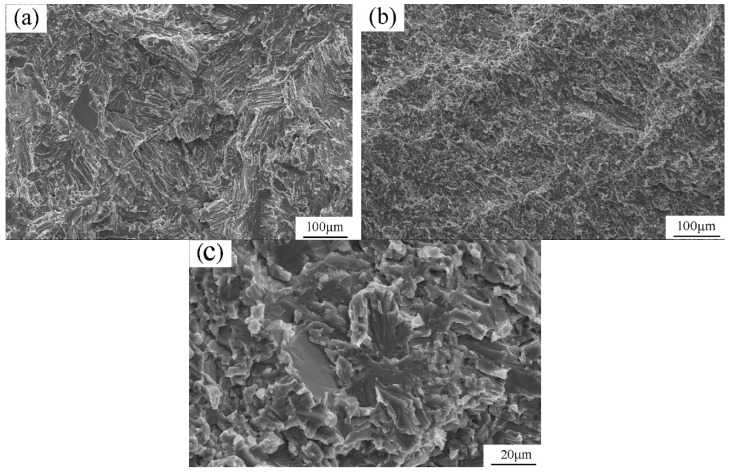
Fractographs of tensile specimens: (**a**) CJ with 150× magnification; (**b**) FJ with 150× magnification; (**c**) FJ with 800× magnification.

Figure 12 shows the fractographs of the CJ and FJ specimens. Observed at 150× magnification, the fractograph of CJ specimen is clear and shows that the predominant fracture mode is translamellar failure with a small amount cleavage planes (Figure 12a). However, the fracture mode of FJ cannot be discerned by the fractograph with 150× magnification (Figure 12b). This is due to the fact that the grain size of the as-forged alloy is an order of magnitude smaller than that of the as-cast alloy. The higher magnification fractograph shows that FJ is a transgranular cleavage-like failure, including translamellar cleavage and delamination (Figure 12c). These distinctive patterns of fracture also show that these specimens failed in a brittle manner.

**Figure 13:**
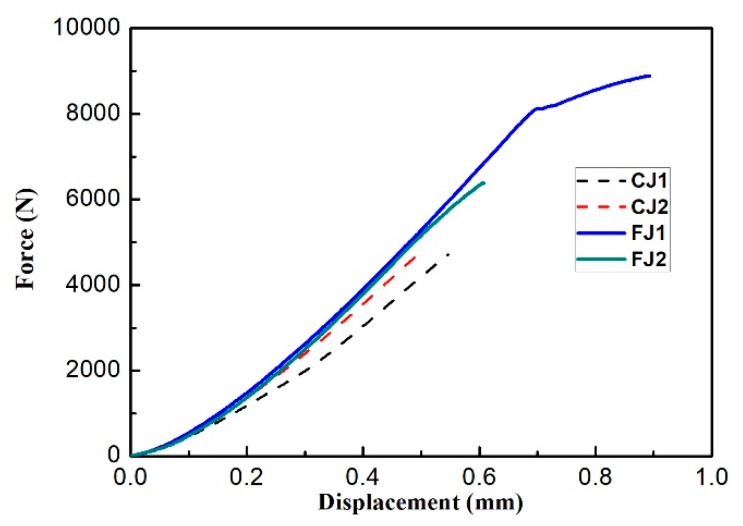
Force-displacement curves of the tensile specimens.

Figure 13 shows the force-displacement curves of the two kinds of joints. The curves of FJ specimens are represented by two solid lines and those of CJ specimens are represented by two thin dotted lines. It can be seen from the curves that the FJ specimen has better strength and ductility than CJ specimen. The displacement includes the displacement of the tensile specimen and displacements of the universal joint and draw rods. Therefore, the plastic elongation (PE) of the specimen is measured by fitting the fractured specimen together and measuring the final elongation with calipers. However, the force values are accurate and the ultimate strength (UTS) of each specimen is calculated by the ratio of the force at fracture to the area of the gauge section. The UTS and PE of each specimen and the corresponding mean value of each kind of specimen are shown in Table 2. For comparison, the UTSs and PEs of the as-cast and the as-forged alloys, which were previously measured with plate specimens by our group, are also shown in the table [24]. The average value of UTS for CJ and FJ specimens are 488MPa and 794 MPa, respectively. Though all the specimens show a brittle fracture mode, the two FJ specimens have the PE of 0.06% and 0.40%, respectively. By contrast, the two CJ specimens have no elongation at all. 

The tensile property of welded specimen depends on the PM because the specimen failed in it. However, both kinds of specimens have worse mechanical properties than their respective PMs. This is due to the difference in experimental conditions between the present work and the previous work, such as the geometry of the tensile specimen, the rate of tensile strain, and the treatment processing of the specimen. Strictly speaking, the results of these two works cannot be compared. These results are listed in the table to show that the present results are consistent with the trend of that the strength and the ductility of the as-cast alloy are lower than those of the as-forged alloy. The increased properties of the as-forged microstructure result partly from the finer grain size of the DP microstructure, leading to more uniform yielding and lower plastic incompatibilities at the grain boundaries during plastic deformation. The same reason can explain why the SDZ and TZ are stronger than the PM regardless of the as-cast and as-forged alloys.

#### 3.5.2. Weldability Assessment

There exist these facts for the as-cast and as-forged alloys: (1) Sound and dense welding joints can be obtained for both as-cast and as-forged alloys, (2) the welding process can be finished without preheating and post-welding heat treatments (PWHT), (3) special pretreatment of contact surface is unnecessary, and (4) the joints are stronger than the PMs. Thus, we can draw a clear conclusion that the present alloy has good weldability from the perspectives of fabrication by RFW and room-temperature strengths of the joints. Our current results show that, at room temperature, the strength of CJ is greater than 480 MPa, while the strength of FJ is greater than 660 MPa. At elevated temperatures grain boundary sliding may play a role in deformation, the joints may be inferior to the PMs in creep properties. Control microstructure of the joint by PWHT may be a valid path to obtain good comprehensive properties. The related works will be further studied in the next step. 

The appearance and the microstructure of CJ and FJ have shown the repeatability of the welding process. However, the reproducibility of tensile properties is also essential. Although the strength of the welds is higher than that of the PMs, it is worthy to note that the tensile properties of the FJ specimens are scattered. This is attributed to the uniformity of the as-forged microstructure, i.e., the volume fraction and morphology of the residual lamellar colony vary in the two specimens. We will improve the microstructure of the as-forged alloy to evaluate the consistency of tensile properties of FJ in the future.

## 4. Conclusions

(1) After trial welding, cylindrical rods of the Ti-45Al-8.5Nb-0.2W-0.2B-0.02Y alloy with both as-cast (NL) and as-forged (DP) microstructures have been successfully welded by rotary friction welding with an optimized process. Each joint has a severe deformation zone (SDZ) with a biconcave lens geometry. The thickness of the SDZ is dozens of micrometers to hundreds of micrometers from the center to periphery. Each joint has a TZ between SDZ and PM, which is hundreds of micrometers in thickness.
(2) Irrespective of the initial microstructure, all SDZs shows a fine DP microstructure with a grain size of a few micrometers due to complete recrystallization occurring in these zones over a short period of time.
(3) The TZ of the joint of the as-cast alloy consists of a large volume fraction of deformed lamellar colonies and a small part of partially recrystallized grains in a NL morphology. This is attributed to the fact that recrystallized grains align along the initial lamellar direction whereas new lamellar colonies keep a similar orientation with the initial one. The retained NL structure has blurred lamellar interfaces. This is owing to the intersection of deformation twins, the lamella growing into adjacent lamellae, and the lamellae thinning by the as-cast lamellae → α → new lamellae transformation.
(4) The TZ of the joint of the as-forged alloy is a DP microstructure. The grain size, the volume fraction of γ grain and the volume fraction of the remnant lamellae all increase with the increase of the distance from SDZ. Full recrystallization occurred in some remnant lamellar colonies.
(5) Under tensile stress, all the joints failed in PMs in brittle fracture mode: CJ mainly showed translamellar failure and FJ showed transgranular cleavage and translamellar failure.
(6) The sound and stronger CJ and FJ show that Ti-45Al-8.5Nb-0.2W-0.2B-0.02Y alloy has good friction weldability.

## Figures and Tables

**Table 1:** Welding parameters used for trial welding.

Scheme	δL (mm)	P_f_ (MPa)	P_u_ (MPa)	$\dot{\varepsilon }$ (mm/s)	ΔL (mm)
1	3	240	300	4	4.35
2	2	240	280	4	2.35
3	3	240	280	4	4.21
4	3	280	280	4	4.50
5	2	320	320	4	3.64
6	3	320	360	4	4.46
7	3	320	360	20	3.98
8	3	360	360	4	-

**Table 2:** Tensile properties of the specimens and the PMs.

	CJ1	CJ2	Mean Value of CJ	As-Cast Alloy [24]	FJ1	FJ2	Mean Value of FJ	As-Forged Alloy [24]
UTS (MPa)	490	486	488	633	924	664	794	897
PE (%)	0	0	0	0.23	0.40	0.06	0.23	2.2

## Abbreviations

BSE	Backscattered electron
CJ	Joint of as-cast alloy
CLSM	Confocal laser microscope
DP	Duplex
EDM	Electrical discharge machining
FJ	Joint of as-forged alloy
LCr	Fine recrystallized lamellar colonies
LCc	Lamellar colonies in as-cast alloy
NL	Nearly lamellar
PE	Plastic elongation at fracture
PM	Parent metal
PWHT	Post-welding heat treatments
RFW	Rotary friction welding
SDZ	Severely deformation zone
SEM	Scanning electron microscope
TZ	Transition zone
TEM	Transmission electron microscope
UTS	Ultimate tensile strength
ω	Rotation speed
P_f_	Friction pressure
δL	Friction burn-off distance
dL	Upset distance
ΔL	Size reduction of the specimen
P_u_	Upset pressure
$\dot{\varepsilon }$	Upset rate
